# Survival from cancer of the pancreas in England and Wales up to 2001

**DOI:** 10.1038/sj.bjc.6604576

**Published:** 2008-09-23

**Authors:** E Mitry, B Rachet, M J Quinn, N Cooper, M P Coleman

**Affiliations:** 1 Département d'Hépatogastroentérologie et Oncologie Digestive, Centre Hospitalo-Universitaire Ambroise-Paré, 9 avenue Charles de Gaulle, F-92100 Boulogne, France; 2 Cancer Research UK Cancer Survival Group, Non-Communicable Disease Epidemiology Unit, Department of Epidemiology and Population Health, London School of Hygiene and Tropical Medicine, Keppel Street, London WC1E 7HT, UK; 3 Social and Health Analysis and Reporting Division, Office for National Statistics (Room FG/114), 1 Myddelton Street, London EC1R 1UW, UK

Cancer of the pancreas accounts for some 3% of cancer in both sexes combined, with about 6000 new cases a year (Cooper *et al*, 2005). The only established risk factor for pancreatic cancer is tobacco smoking (Lowenfels, 1984). Incidence has fallen some 10–15% since the mid-1970s in men but has risen slightly in women, and annual incidence in both sexes is now similar at about 10 per 100 000 (Quinn *et al*, 2001). Both incidence and mortality rates may be biased, however, by under-registration of incident cases and an over-certification of deaths (Remontet *et al*, 2003). In this context, the survival of patients who were registered with a diagnosis of pancreatic cancer in life assumes particular importance as a measure of outcome. Pancreatic cancer has one of the worst prognoses: the European mean relative 5-year survival rate for patients diagnosed during 1990–1994 was less than 4% (Sant *et al*, 2003).

We analysed the data for 62 815 patients registered with pancreatic cancer in England and Wales during the period 1986–1999, only 74% of those were apparently eligible. More than a fifth (22%) of all cases had to be excluded from survival analysis because their recorded survival was zero (date of diagnosis same as date of death): some will in fact have died on the day of diagnosis, but most were registered from a death certificate only (DCO), and their survival time was unknown. Some 3% of cases in the national data were known to have been DCOs, but the proportion varied by registry, and they could not be reliably distinguished from cases with true zero survival in the national data. The proportion of cases whose recorded survival was zero rose from 12 to 14% in the 1970s to 19–22% in the 1990s (Coleman *et al*, 1999). As they represent such a large proportion of patients who were otherwise eligible for survival analysis, and who may have shorter than average survival (Berrino *et al*, 1995), the impact of their exclusion on observed trends and inequalities in survival needs to be considered. Nationally, the proportion of such cases fluctuated slightly within the range 19–25%. Trends in the proportion of such cases, however, varied very widely among regions: stable and relatively low (6–11%) in Northern and Yorkshire, East Anglia and Oxfordshire; initially high but falling rapidly (Thames, Wales); or initially low but rising rapidly (South and West, West Midlands). These patterns are hard to interpret, as the efficiency and timeliness of registration have increased over this period, and the proportion of other cancers registered solely from a death certificate has generally declined. Overall estimates of survival may thus be slightly high. In contrast, the impact of exclusions for zero survival on trends in the deprivation gap in survival during the 1990s is likely to have been small or negligible, as there was no systematic difference in the proportion of such cases among socioeconomic groups (data not shown). The vital status of 1.4% of patients was unknown at 5 November 2002, and a further 2.8% were also excluded because pancreatic cancer was not their first primary malignancy.

## Survival trends

**Figure 1:**
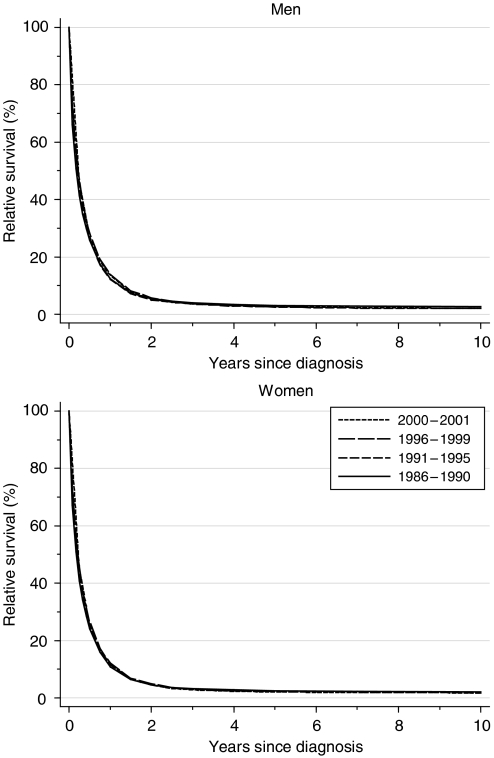
Relative survival (%) up to 10 years after diagnosis by sex and calendar period of diagnosis: England and Wales, adults (15–99 years) diagnosed during 1986–1999 and followed up to 2001. Survival estimated with cohort or complete approach (1986–1990, 1991–1995, 1996–1999) or hybrid approach (2000–2001) (see Rachet *et al*, 2008).

Short-term survival has increased slightly in men, but there is no evidence of improvement in longer-term survival in either sex (Figure 1). One-year survival rose slightly from around 12% for men diagnosed during the 10 years 1986–1995 to 13.9% for those diagnosed during 1996–1999. The fitted, deprivation-adjusted average increase of 1.6% every 5 years was statistically significant (Table 1). For women, 1-year survival rose very slowly, from 10.9 to 12.1%, with an average increase of just 0.3% every 5 years. There was, however, no improvement at all in 5-year survival, which was actually slightly lower for those diagnosed during 1996–1999 (2.7% in men, 2.3% in women) than for those diagnosed during 1986–1990 (3.1 and 2.5%, respectively). Median survival is less than 6 months in both sexes, and it did not improve.

Short-term predictions of survival for patients diagnosed during 2000–2001, using hybrid analysis (Brenner and Rachet, 2004), show no evidence of any pending improvement in survival up to 10 years after diagnosis (Table 1).

## Deprivation

**Figure 2:**
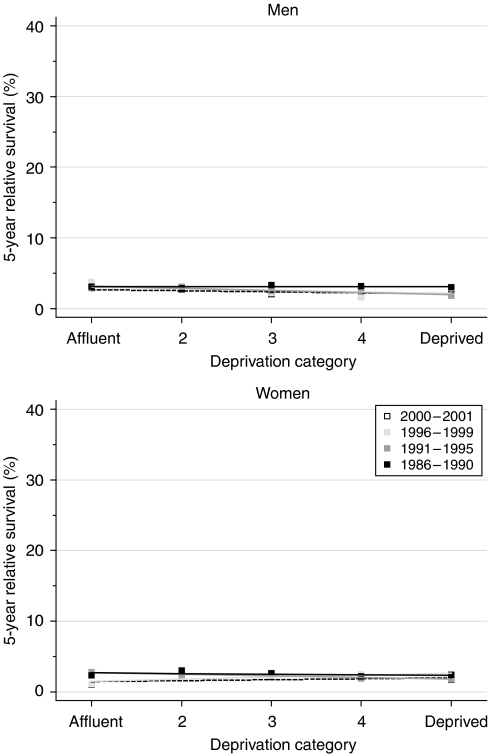
Trends in the deprivation gap in 5-year relative survival (%) by sex and calendar period of diagnosis: England and Wales, adults (15–99 years) diagnosed during 1986–1999 and followed up to 2001.

Differences in survival between deprivation categories were small. For those diagnosed during the late 1990s, the deprivation gap in 1- and 5-year survival was slightly more marked in men (Table 2, Figure 2). For women diagnosed in 1996–1999, 5-year survival was slightly better in the deprived than the affluent (deprivation gap positive, +1.1%) and this difference was of borderline statistical significance. The deprivation gap in survival for women diagnosed during the 14 years 1986–1999 appears to have fallen for 1-year survival or even reversed for 5-year survival, but the fitted average improvement every 5 years was less than 1%, and this trend was not statistically significant. There was no systematic trend towards better survival in the deprived than the affluent, and short-term predictions of survival for patients diagnosed during 2000–2001, using hybrid analysis, do not suggest any imminent change in the deprivation gap.

## Comment

Relative survival at 5 years, less than 3% in both sexes, is lower than for any other of the 20 most common adult cancers in England and Wales. No improvement has occurred since the 1970s (Coleman *et al*, 1999). Despite progress in diagnostic procedures, most cases are still metastatic at diagnosis, and are not amenable to surgical resection of curative intent. Even when curative surgery can be attempted, most patients will relapse. A small trial has shown that palliative chemotherapy may extend median survival from 2.5 to 6 months (Glimelius *et al*, 1996), but efficacy remains disappointing, and any therapeutic progress over the last 30 years has not been reflected in higher survival for all patients with pancreatic cancer.

## Figures and Tables

**Table 1:** Trends in relative survival (%) by sex, time since diagnosis and calendar period of diagnosis: England and Wales, adults (15–99 years) diagnosed during 1986–1999 and followed up to 2001

		**Calendar period of diagnosis^a^**						**Average change (%)**		**Prediction^c^ for patients**	
		**1986–1990**		**1991–1995**		**1996–1999**		**every 5 years^b^**		**diagnosed during 2000–2001**	
**Time since diagnosis**		**Survival (%)**	**95% CI**	**Survival (%)**	**95% CI**	**Survival (%)**	**95% CI**	**Survival (%)**	**95% CI**	**Survival (%)**	**95% CI**
1 year	Men	**12.4**	(11.8, 13.0)	**12.2**	(11.6, 12.8)	**13.9**	(13.2, 14.6)	**1.6** ^*^	(0.2, 3.0)	**13.6**	(12.6, 14.6)
	Women	**10.9**	(10.3, 11.5)	**11.6**	(11.0, 12.2)	**12.1**	(11.5, 12.8)	**0.3**	(−0.9, 1.6)	**11.5**	(10.6, 12.5)
5 years	Men	**3.1**	(2.8, 3.5)	**2.5**	(2.2, 2.8)	**2.7**	(2.3, 3.1)	**0.1**	(−0.6, 0.9)	**2.6**	(2.2, 3.2)
	Women	**2.5**	(2.2, 2.8)	**2.2**	(1.9, 2.5)	**2.3**	(1.9, 2.7)	−0.8^*^	(−1.4, −0.1)	**2.0**	(1.6, 2.5)
10 years	Men	**2.6**	(2.3, 3.0)	**2.0**	(1.7, 2.3)			**0.6**	(−0.7, 1.9)	**2.1**	(1.7, 2.7)
	Women	**2.1**	(1.8, 2.4)	**1.8**	(1.5, 2.1)			**0.5**	(−0.7, 1.7)	**1.8**	(1.4, 2.2)

CI=confidence interval.

a Survival estimated with cohort or complete approach (see Rachet *et al*, 2008).

b Mean absolute change (%) in survival every 5 years, adjusted for deprivation (see Rachet *et al*, 2008).

c Survival estimated with hybrid approach (see Rachet *et al*, 2008).

^*^P<0.05.

**Table 2:** Trends in the deprivation gap in relative survival (%) by sex, time since diagnosis and calendar period of diagnosis: England and Wales, adults (15–99 years) diagnosed during 1986–1999 and followed up to 2001

		**Calendar period of diagnosis^a^**						**Average change (%)**		**Prediction^c^ for patients**	
		**1986–1990**		**1991–1995**		**1996–1999**		**every** **5 years^b^**		**diagnosed during 2000–2001**	
**Time since diagnosis**		**Deprivation gap (%)**	**95% CI**	**Deprivation gap (%)**	**95% CI**	**Deprivation gap (%)**	**95% CI**	**Deprivation gap (%)**	**95% CI**	**Deprivation gap (%)**	**95% CI**
1 year	Men	−**0.9**	(−2.7, 1.0)	−3.2^**^	(−5.0, −1.4)	−2.8^*^	(−4.9, −0.6)	−**1.1**	(−2.6, 0.4)	−3.4^*^	(−6.4, −0.5)
	Women	−**1.5**	(−3.3, 0.2)	−3.5^**^	(−5.3, −1.8)	−**0.5**	(−2.5, 1.4)	**0.4**	(−1.0, 1.7)	−**1.0**	(−3.7, 1.7)
5 years	Men	**0.0**	(−1.0, 1.1)	−1.2^**^	(−2.1, −0.3)	−**0.9**	(−2.2, 0.4)	−**0.6**	(−1.5, 0.3)	−**0.6**	(−2.0, 0.8)
	Women	−**0.4**	(−1.3, 0.6)	−0.9^*^	(−1.8, −0.1)	1.1^*^	(0.1, 2.1)	**0.7**	(0.0, 1.4)	**0.5**	(−0.5, 1.5)
10 years	Men	**0.7**	(−0.3, 1.7)	−**0.8**	(−1.8, 0.1)			−1.5^*^	(−2.9, −0.1)	−**0.5**	(−2.0, 0.9)
	Women	**0.2**	(−0.8, 1.1)	−0.9^*^	(−1.8, 0.0)			−**1.1**	(−2.3, 0.2)	**0.5**	(−0.4, 1.5)

CI=confidence interval.

a Survival estimated with cohort or complete approach (see Rachet *et al*, 2008).

b Mean absolute change (%) in the deprivation gap in survival every 5 years, adjusted for the underlying trend in survival (see Rachet *et al*, 2008).

c Survival estimated with hybrid approach (see Rachet *et al*, 2008).

^*^ *P*<0.05; ^**^P<0.01.

## References

[bib1] Berrino F, Estève J, Coleman MP (1995) Basic issues in the estimation and comparison of cancer patient survival. In Survival of Cancer Patients in Europe: the EUROCARE Study. (IARC Scientific Publications No. 132). Berrino F, Sant M, Verdecchia A, Capocaccia R, Hakulinen T, Estève J (eds), pp 1–14. International Agency for Research on Cancer: Lyon

[bib2] Brenner H, Rachet B (2004) Hybrid analysis for up-to-date long-term survival rates in cancer registries with delayed recording of incident cases. Eur J Cancer 40: 2494–2501 15519525 10.1016/j.ejca.2004.07.022

[bib3] Coleman MP, Babb P, Damiecki P, Grosclaude PC, Honjo S, Jones J, Knerer G, Pitard A, Quinn MJ, Sloggett A, De Stavola BL (1999) Cancer Survival Trends in England and Wales 1971–1995: Deprivation and NHS Region. Studies on Medical and Population Subjects No. 61. The Stationery Office: London

[bib4] Cooper N, Gautrey M, Quinn MJ (2005) First release: Cancer: number of new cases, 2002. Office for National Statistics. http://www.statistics.gov.uk/StatBase/

[bib5] Glimelius B, Hoffman K, Sjoden PO, Jacobsson G, Sellstrom H, Enander LK, Linne T, Svensson C (1996) Chemotherapy improves survival and quality of life in advanced pancreatic and biliary cancer. Ann Oncol 7: 593–600 8879373 10.1093/oxfordjournals.annonc.a010676

[bib6] Lowenfels AB (1984) Chronic pancreatitis, pancreatic cancer, alcohol and smoking. Gastroenterology 87: 744–745 6745623

[bib7] Quinn MJ, Babb P, Brock A, Kirby L, Jones J (2001) Cancer Trends in England and Wales 1950–1999. Studies on Medical and Population Subjects No. 66. Office for National Statistics: London

[bib8] Remontet L, Estève J, Bouvier A-M, Grosclaude PC, Launoy G, Ménégoz F, Tretarre B, Exbrayat C, Carli P-M, Guizard AV, Troussard X, Bercelli P, Colonna M, Halna JM, Hédelin G, Macé-Lesech J, Peng J, Buémi A, Velten M, Jougla E, Arveux P, Le Bodic L, Michel E, Sauvage M, Schwartz J, Faivre J (2003) Cancer incidence and mortality in France over the period 1978–2000. Rev épidémiol santé publique 51: 3–30 12684578

[bib9] Rachet B, Woods LM, Mitry E, Riga M, Cooper N, Quinn MJ, Steward J, Brenner H, Estève J, Sullivan R, Coleman MP (2008) Cancer survival in England and Wales at the end of the 20th century. Br J Cancer 99(Suppl 1): S2–S10 18813248 10.1038/sj.bjc.6604571 PMC2557545

[bib10] Sant M, Aareleid T, Berrino F, Bielska Lasota M, Carli P-M, Faivre J, Grosclaude PC, Hédelin G, Matsuda T, Møller H, Moller T, Verdecchia A, Capocaccia R, Gatta G, Micheli A, Santaquilani M, Roazzi P, Lisi D, EUROCARE Working Group (2003) EUROCARE-3: survival of cancer patients diagnosed 1990–94 – results and commentary. Ann Oncol 14(Suppl 5): 61–118 10.1093/annonc/mdg754 14684501

